# Impact of Respiratory Syncytial Virus and Severe Acute Respiratory Syndrome Coronavirus 2 Coinfection on Clinical Severity and Outcomes Among Children Hospitalized With Lower Respiratory Tract Infections in Soweto, South Africa

**DOI:** 10.1097/INF.0000000000004560

**Published:** 2024-10-02

**Authors:** Kitso-Lesedi Mrubata, Vicky Bailie, Fatima Solomon, Alane Izu, Musawenkosi Ncube, Marta C. Nunes, Ziyaad Dangor, Shabir A. Madhi, David P. Moore, Charl Verwey

**Affiliations:** From the * Department of Paediatrics and Child Health, Faculty of Health Sciences, School of Clinical Medicine, University of the Witwatersrand, Johannesburg, South Africa; † South African Medical Research Council Vaccines and Infectious Diseases Analytics Research Unit, University of the Witwatersrand, Johannesburg, South Africa; ‡ Department of Science, Faculty of Health Science, National Research Foundation: Vaccine Preventable Diseases, University of the Witwatersrand, Johannesburg, South Africa; § Center of Excellence in Respiratory Pathogens (CERP), Hospices Civils de Lyon (HCL) and Centre International de Recherche en Infectiologie (CIRI), Équipe Santé Publique, Épidémiologie et Écologie Évolutive des Maladies Infectieuses (PHE3ID), Université Claude Bernard Lyon 1, Lyon, France.

**Keywords:** respiratory syncytial virus, SARS-CoV-2, lower respiratory tract infection

## Abstract

**Background:**

No data are available regarding the interplay and clinical manifestations of respiratory syncytial virus (RSV) and severe acute respiratory syndrome coronavirus-2 (SARS-CoV-2) coinfection in African children. We compared clinical characteristics and outcomes between RSV-only, SARS-CoV-2–only and RSV/SARS-CoV-2 coinfection lower respiratory tract infections (LRTI) in hospitalized African children.

**Methods:**

Prospective surveillance of children (0–59 months) hospitalized with severe LRTI was undertaken between March 1, 2020, and March 31, 2023, in Johannesburg, South Africa. Nasopharyngeal swabs for respiratory viruses and clinical data were collected, and clinical characteristics and outcomes were described and compared. Respiratory index of severity in children (RISC) scores were calculated for HIV-uninfected children, and covariates associated with high RISC scores (≥5) were evaluated.

**Results:**

Seven thousand four hundred fifty-six children [6.1 months (interquartile range, 14.4–18.6); 57.7% male] were enrolled, 1372 (18.4%) testing RSV+/SARS-CoV-2– (RSV only), 223 (3.0%) RSV−/SARS-CoV-2+ (SARS-CoV-2–only) and 28 (0.4%) RSV+/SARS-CoV-2+ (RSV/SARS-CoV-2 coinfection). Children with RSV only and RSV/SARS-CoV-2 coinfection were more likely to present with bronchiolitis than those with SARS-CoV-2–only (673/1372 and 15/28 vs. 46/223; *P* < 0.001). Children with RSV/SARS-CoV-2 coinfection had more severe disease than those with RSV or SARS-CoV-2–only, as well as a higher RISC score than SARS-CoV-2–only. Weight-for-age *Z* scores [adjusted risk ratio (aRR): 0.92], room air saturations (aRR: 0.988) and RSV+ status (aRR: 1.40) were independently associated with severe disease.

**Conclusions:**

Although both RSV and SARS-CoV-2 LRTI occurred commonly, coinfection did not. Children with RSV/SARS-CoV-2 coinfection had a higher prevalence of severe LRTI than those with RSV or SARS-CoV-2–only. These findings reinforce the urgent need for safe and effective RSV and SARS-CoV-2 vaccines, especially in children in low- and middle-income countries, where the burden of disease is the highest and the access to medical resources the lowest.

## INTRODUCTION

Lower respiratory tract infections (LRTI) are a major cause of under-5 childhood mortality outside of the neonatal period, accounting for 12% of all deaths, with the majority occurring in low- and middle-income countries (LMIC).^1^ Viral pathogens are the most common cause of LRTI, with respiratory syncytial virus (RSV) the leading pathogen, accounting for approximately a third of all LRTI in hospitalized children and 101,000 annual deaths globally.^2^ Even though vaccines targeting multiple causes of childhood LRTI, such as *Haemophilus influenzae* type b, pneumococcal conjugate, measles, influenza and *Bordetella pertussis*, have dramatically reduced the incidence of LRTI, the emergence of successful candidate immunization strategies targeting RSV are only recently becoming available.^3^

The first cases of severe LRTI attributed to severe acute respiratory syndrome coronavirus 2 (SARS-CoV-2) were identified in Wuhan, China, in December 2019, with the first confirmed case in South Africa reported on March 5, 2020. The World Health Organization subsequently designated the disease coronavirus disease 2019 (COVID-19) and declared a pandemic in March 2020.^4,5^ There have been over 770 million COVID-19 cases and approximately 6.9 million deaths worldwide, with South Africa accounting for 4.0 million confirmed cases and over 102,000 deaths.^6^ Multiple variants and lineages, mostly arising from mutations in the spike protein, have been identified, with variants of significance being alpha, beta, gamma, delta and omicron.^4,5^

Fever is the most common symptom in pediatric patients with COVID-19, along with upper respiratory tract symptoms or mild gastrointestinal symptoms.^7^ Clinical manifestations in pediatric patients during the first to third waves of the COVID-19 pandemic remained similar among children in South Africa.^8^ During the fourth wave (which was dominated by the Omicron variant), fever, cough, vomiting, difficulty breathing, diarrhea and seizures being prominent clinical features.^8^

RSV and SARS-CoV-2 coinfection may have important implications on the morbidity and mortality of pediatric patients. Existing knowledge and data have explored each of these infections individually within the pediatric population; however, there is a paucity of data describing the clinical characteristics and patient outcomes of children with RSV/SARS-CoV-2 coinfection, and none from LMICs. Rodriguez-Fernandez et al^9^ described 57 children less than 2 years of age from Spain, between March 2020 and January 2023, admitted to hospital with RSV bronchiolitis, and reported that only 2.1% had coinfection with SARS-CoV-2. Children with coinfection were reported to require a prolonged length of hospital stay and were more likely to require high-flow nasal cannula oxygen support.^9^ The aim of this study was to compare clinical characteristics and outcomes of RSV or SARS-CoV-2–only to RSV/SARS-CoV-2 coinfections in children (0–59 months) hospitalized for acute severe LRTI.

## MATERIALS AND METHODS

We conducted a prospective surveillance study comparing the demographics, clinical characteristics and outcomes of RSV or SARS-CoV-2–only and RSV/SARS-CoV-2 coinfected children 0–59 months of age, hospitalized for severe acute LRTI at Chris Hani Baragwanath Academic Hospital, a tertiary academic hospital, in Johannesburg, South Africa, from January 1, 2020, to March 31, 2023. Acute severe LRTI was defined as acute onset of cough (less than 7 days), or difficulty breathing with lower chest wall indrawing with or without fast breathing.^10^ The diagnosis of bronchiolitis or bronchopneumonia was made by the attending doctor at the time of presentation, who was either a general practitioner, a pediatric trainee, or a pediatrician. Chest radiographs were not routinely performed; therefore, the diagnosis was based on clinical findings.

LRTI surveillance in children under 5 years of age has been routinely conducted at Chris Hani Baragwanath Academic Hospital since 2014. Children admitted with severe LRTI and consented have nasopharyngeal swabs (FLOQS, Copan Flock Technologies, Brescia, Italy) collected within 72 hours of admission. Swabs are collected from all participants between Monday and Friday, as well as those admitted over weekends, who were still admitted the following Monday. These swabs are processed at the Vaccines and Infectious Diseases Analytics Research Unit laboratory, where total nucleic acid extraction using the automated NucliSENS EasyMAG (BioMérieux, Marcy l’Etoile, France) nucleic acid extraction method was performed. A 1-step multiplex polymerase chain reaction assay developed in-house, detecting RSV A, RSV B, human metapneumovirus, influenza A and B and *B. pertussis* (targeting IS481) was used in this study; only RSV results are reported in this analysis. A cycle threshold value of <37 was used as a cutoff for identifying positive RSV samples. Data detailing demographics, risk factors and clinical data from all cases were prospectively collected on all study participants.

All children hospitalized after March 2020 had a second nasopharyngeal specimen tested for SARS-CoV-2 using emergency use authorization assays developed by the Centers for Diseases Control and Prevention, which target 2 regions within the nucleocapsid gene.^11^ After the emergence of the Omicron variant (November 2021), testing transitioned to TaqPath COVID-19 assays (Thermo Fisher Scientific, Pleasanton, CA). Total nucleic acids were extracted from the nasopharyngeal swabs using the Bioer automated extraction system together with MagaBio plus Virus DNA/RNA purification kit II as per manufacturers’ instructions (Hangzhou Bioer Technology Co. Ltd, China).

HIV testing was performed on children according to the South African National HIV testing guidelines. For this study, children HIV-exposed uninfected and unexposed uninfected, were grouped together as HIV uninfected.

Categorical data were described using frequencies and percentages, and continuous data using means and standard deviation for parametric variables and medians and interquartile range (IQR) for nonparametric. Categorical data were compared using Chi-squared or Fisher exact test and continuous data using unpaired *t* tests or the Wilcoxon rank sum test, and summary statistics were reported with *P* values. Multivariable Poisson regression analysis was undertaken to describe any association between variables and a composite index severity score, defined by hospitalization >3 days, invasive ventilation required, or death in hospital. Furthermore, a respiratory index of severity in children (RISC) score was assigned to HIV-uninfected children with available data (oxygen saturation, presence of chest indrawing, wheezing, refusal of feeds, and weight-for-age *Z* score).^12^ RISC scores for HIV-infected children could not be derived, as no data on the World Health Organization Clinical Stage of HIV disease was captured in the database. Variable selection for multivariable Poisson regression models was undertaken using least absolute shrinkage and selection operator regression. All analyses were conducted using R version 4.4.0 (R Core Team, Austria).

Ethics approvals were obtained through the University of the Witwatersrand Human Resources Ethics Committee (M140904 and M220643).

## RESULTS

From March 1, 2020, to March 31, 2023, 7456 children [6.1 months (IQR, 14.4–18.6); 57.7% male] were tested as part of LRTI and SARS-CoV-2 surveillance protocols in children under 5 years of age. Five thousand eight hundred and thirty-three (78.2%) tested negative for both RSV and SARS-CoV-2. One thousand three hundred and seventy-two (18.4%) tested RSV+/SARS-CoV-2− (RSV-only), 223 (3.0%) RSV−/SARS-CoV-2+ (SARS-CoV-2–only) and 28 (0.4%) RSV+/SARS-CoV-2+ (RSV/SARS-CoV-2 coinfection) (Table 1). RSV/SARS-CoV-2 coinfection was noted in each year of the study, with 1 case (0.6%) of 158 children tested identified in 2020, 13 (0.5%) of 2503 of children tested in 2021, 8 (0.2%) of 3346 tested children in 2022 and 6 (0.4%) from 1449 children tested in 2023.

**TABLE 1.** Characteristics of Children, Stratified by RSV and SARS-CoV-2 Infection Status

	A	B	C	A vs. B	B vs. C	A vs. C
	RSV+/SARS-CoV-2- (n = 1372)	RSV+/SARS-CoV-2+ (n = 28)	RSV−/SARS-CoV-2+ (n = 223)	*P* Value	*P* Value	*P* Value
Demographics						
Median age (mo, IQR)	3.91 (1.73–10.90)	3.64 (1.40–6.14)	4.80 (1.46–11.87)	0.341	0.195	0.326
Male (%)	744 (54.2)	19 (67.9)	117 (52.5)	0.214	0.180	0.677
Any comorbidity (%)	63 (4.6)	0 (0.0)	22 (9.9)	0.484	0.166	**0.002**
Weight-for-age *Z* score (median, IQR)	−0.90 (−1.93 to 0.12)	−0.46 (−1.98 to 0.63)	−1.23 (−2.94 to −0.16)	0.343	**0.048**	**0.002**
Weight-for-length *Z* score (median, IQR)	0.28 (−1.00 to 1.39)	0.80 (−0.30 to 1.50)	0.02 (−1.26 to 1.13)	0.327	0.100	0.053
Clinical indices						
Admission respiratory rate (median, IQR)	48 (40–60)	54 (46–62)	42 (34–52)	0.093	**<0.001**	**<0.001**
Temperature on admission (°C) (median, IQR)	36.9 (36.5–37.4)	36.9 (36.5–37.2)	36.9 (36.5–37.5)	0.857	0.951	0.573
Coryza (%)	410 (30.8)	9 (33.3)	54 (24.2)	0.945	0.426	0.055
Nasal flaring (%)	494 (36.2)	7 (25.9)	52 (23.4)	0.368	0.961	**<0.001**
Grunting (%)	77 (5.6)	0 (0.0)	10 (4.5)	0.399	0.542	0.607
Vomiting (%)	472 (35.1)	8 (29.6)	54 (24.4)	0.700	0.724	**0.002**
Diarrhea (%)	245 (18.2)	7 (25.9)	55 (24.9)	0.442	1.000	**0.026**
Abdominal pain (%)	21 (2.2)	0 (0.0)	13 (6.4)	1.000	0.479	**0.003**
Rash (%)	35 (3.7)	1 (4.8)	7 (3.5)	1.000	1.000	1.000
Respiratory distress (%)	1067 (77.9)	21 (77.8)	106 (47.7)	1.000	**0.006**	**<0.001**
Wheeze (%)	496 (36.4)	10 (37.0)	34 (15.4)	1.000	**0.012**	**<0.001**
Crackles (%)	702 (51.5)	14 (51.9)	56 (25.3)	1.000	**0.008**	**<0.001**
Diagnosis						
Bronchopneumonia (%)	491 (39.0)	12 (48.0)	73 (34.0)	0.478	0.242	0.186
Bronchiolitis (%)	673 (53.4)	14 (56.0)	46 (21.4)	0.957	**<0.001**	**<0.001**
Indices of severity						
Admission room air saturations (%) (median, IQR)	86 (1–93)	87 (75–92)	96 (90–98)	0.555	**<0.001**	**<0.001**
Admission saturations on supplemental oxygen (%) (median, IQR)	95 (1–98)	95 (92–96)	97 (89–99)	0.927	0.120	**0.018**
Ventilated (%)	17 (1.4)	1 (4.2)	2 (1.0)	0.459	0.074	0.175
Length of hospitalization (d) (median, IQR)	3 (0–5)	4 (3–6.5)	3 (2–6)	0.041	0.325	**0.001**
Died in-hospital (%)	23 (1.7)	1 (3.7)	8 (3.6)	0.961	1.000	0.100
Index of severity (%)	588 (45.8)	18 (72.0)	103 (47.2)	**0.017**	**0.033**	0.753
RISC score ≥5 (%)	130 (15.4)	3 (15.8)	11 (6.3)	1.000	**0.002**	0.296

The numbers in bold are those that are statistically significant (*P* < 0.05).

Index of severity assigned to children with either hospitalization >3 d, or invasive ventilation required, or death in hospital. RISC score assigned to HIV-uninfected children with available data (oxygen saturation, presence of chest indrawing, wheezing, refusal of feeds, and weight-for-age *Z* score) in whom RISC scores could be calculated.

There were no differences in age, sex, clinical characteristics or outcomes between the RSV/SARS-CoV-2 coinfection and the RSV-only group (comparison A vs. B; Table 1). However, children in the RSV/SARS-CoV-2 coinfection group had a longer duration of hospitalization (4 vs. 3 days), as well as a higher prevalence of severe disease (as apportioned using the composite index of severity: 72.0% vs. 45.8%) compared with RSV-only.

Children in the RSV/SARS-CoV-2 coinfection group (comparison B vs. C; Table 1) had more severe respiratory disease than those with SARS-CoV-2–only, with an increased respiratory rate at admission (54 vs. 41.5 breaths per minute; *P* < 0.001), increased prevalence of respiratory distress (77.8% vs. 47.7%; *P* = 0.006) and lower oxygen saturations on room air at admission (87% vs. 96%; *P* < 0.001). They also had an increased prevalence of wheezing (37% vs. 15%; *P* = 0.012) or crackles (52% vs. 25%; *P* = 0.008) on auscultation. Outcomes, including mortality and duration of hospital stay were similar between the 2 groups.

Children with RSV-only compared with those with SARS-CoV-2–only (comparison A vs. C; Table 1) at admission had an increased respiratory rate (48 vs. 41.5 breaths per minute; *P* < 0.001), higher prevalence of respiratory distress (78% vs. 48%; *P* < 0.001) and decreased room air oxygen saturation (86% vs. 96%; *P* < 0.001). They also had a higher prevalence of wheezing (36% vs. 15%; *P* < 0.001) and crackles (52% vs. 25%; *P* < 0.001) on auscultation. Children in the SARS-CoV-2 group were more likely to present with abdominal symptoms, including diarrhea (25% vs. 18%; *P* = 0.026) and abdominal pain (6% vs. 2%; *P* = 0.003).

Children with RSV-only or RSV/SARS-CoV-2 coinfection were more likely to be diagnosed with bronchiolitis than those with SARS-CoV-2–only (*P* < 0.001), while there was no difference in bronchiolitis diagnosis between the RSV-only and RSV/SARS-CoV-2 coinfection groups (*P* = 0.957).

Median RISC scores were 2 (IQR, 0–3) in 845 HIV-uninfected children with RSV-only, and 0 (IQR, 0–2) in 174 children with SARS-CoV-2–only (*P* < 0.001). Furthermore, 130 of 859 (15.1%) with RSV-only compared with 11 of 184 (6.0%) with SARS-CoV-2–only had RISC scores ≥5 (*P* = 0.002) (Table 1). Variables that were independently associated with RISC scores ≥5 included weight-for-age *Z* score [adjusted risk ratio (aRR) (95% confidence interval {CI}): 0.919 (0.869–0.969)], room air saturations on admission [aRR (95% CI): 0.988 (0.981–0.996)] and RSV-positive status [aRR (95% CI): 1.402 (1.098–1.784); Table 2].

**TABLE 2.** Multivariable Poisson Regression Model to Identify Factors Independently Associated With High RISC Scores in HIV-uninfected Children (n = 882)

	Univariable Outputs				Multivariable Model			
Parameter	Risk Ratio	Lower Estimate	Upper Estimate	*P* Value	Adjusted Risk Ratio	Lower Estimate	Upper Estimate	*P* value
Intercept	-	-	-	-	0.593	0.294	1.098	0.119
Age (mo)	0.993	0.984	1.000	0.106	0.997	0.987	1.006	0.493
Weight-for-age *Z* score	0.907	0.855	0.961	0.001	0.919	0.869	0.969	**0.003**
Admission respiratory rate	1.000	1.000	1.000	0.090	1.000	1.000	1.001	0.083
Saturations in room air (%)	0.987	0.980	0.994	<0.001	0.988	0.981	0.996	**0.002**
Any comorbidity	1.41	0.957	1.99	0.068	1.433	0.932	2.127	0.086
SARS-CoV-2 +	1.22	0.677	2.01	0.472	1.054	0.542	1.834	0.864
RSV +	1.39	1.11	1.73	0.004	1.402	1.098	1.784	**0.006**

The numbers in bold are those that are statistically significant (*P* < 0.05).

## DISCUSSION

In this observational study, we report data on 1372 children (0–59 months) hospitalized with severe RSV, SARS-CoV-2 or RSV/SARS-CoV-2 coinfection LRTI. Children with RSV or RSV/SARS-CoV-2 coinfection had an increased respiratory rate, more marked respiratory distress and lower oxygen saturations than those with SARS-CoV-2–only. RSV/SARS-CoV-2 coinfection was associated with more severe disease, as defined by length of hospital stay, invasive ventilation requirements and in-hospital mortality, as has been noted by others, and was associated with high RISC scores (≥5), in HIV-uninfected children.^13–15^ Of note RSV infection (either RSV-only or RSV/SARS-CoV-2 coinfection) was independently associated with increased adjusted risk of high RISC scores (≥5).

The presence of wheezing and crackles on chest auscultation was more commonly encountered in children with RSV (only or as a coinfection with SARS-CoV-2) and were more likely to be diagnosed with bronchiolitis, then those with SARS-CoV-2–only.

On univariable analysis, clinical features associated with severe disease included age, nutritional status, oxygen saturation levels on admission and underlying comorbidities, all of which are known associations for severe RSV LRTI.^16^ In a retrospective study from Spain, RSV/SARS-CoV-2 coinfection was associated with longer hospital stay, and a 4.8-fold higher requirement for treatment with high-flow nasal cannula oxygen.^9^ A possible explanation for less severe disease in SARS-CoV-2 mono-infection, compared with RSV/SARS-CoV-2 coinfection, may be a more efficient host immunological response toward SARS-CoV-2.^16,17^

Due to the implementation of nonpharmacological interventions, many studies including ours showed very low rates of RSV infection during the initial phases of the COVID-19 pandemic.^18^ In their study, Uhteg et al,^13^ reviewing the circulation of non-SARS-CoV-2 viruses during the COVID-19 pandemic, highlighted variable circulation of respiratory viruses, which was attributed to differences in viral structure and survival of viral particles on fomites, with RSV forming part of the less frequently detected group. We previously demonstrated a significant 48% reduction in RSV-associated LRTI in 2020, with rebound of RSV-associated LRTI to prepandemic levels in 2022 at our institution.^18^

Through prospective surveillance of respiratory viruses, we detected a low prevalence (28 of 7456; 0.4%) of RSV/SARS-CoV-2 coinfection, which occurred in each year of the study albeit with particularly low prevalence in 2020, probably because of strict lock-down restrictions in the early stages of the pandemic. RSV/SARS-CoV-2 coinfection, although low in studies conducted in the USA and Italy, ranging between 7% and 14% of all tested pediatric LRTI episodes, was much higher than reported by us.^13,14^ Low rates of coinfection may be due to low rates of RSV and SARS-CoV-2–only infection overall, but could also allude to a viral–viral interaction leading to a reduced presentation of LRTI.^16^

Children with any comorbidity were more likely to be admitted for SARS-CoV-2–only infection than for RSV-only infection.^19^ Many studies have reported an increased likelihood of severe disease in children with comorbidities admitted with RSV infection during infancy. This result will further highlight the severe outcomes of children with comorbidities and any LRTI, including SARS-CoV-2 infection.

The strengths of our study include that it consists of prospectively collected data from a large cohort of children hospitalized with respiratory illness, with representation from multiple waves of the COVID-19 pandemic, and over 3 RSV seasons, in South Africa. We were able to evaluate RSV/SARS-CoV-2 coinfection, RSV-only infection and SARS-CoV-2–only infection in stages of the pandemic that had varying lock-down restrictions.

A limitation of our study is that we did not review the effect of other infections, both respiratory viral and/or bacterial, which may have played a role in clinical presentation of patients included in the study. Other viral coinfections, such as coinfection with adenovirus, could have influenced the results, resulting in more severe disease and worse outcomes, however, testing for adenovirus was not being routinely performed. Testing for influenza A, influenza B and human metapneumovirus was routinely performed, but the number of positive cases were very low (influenza A, n = 7; influenza B, n = 0; human metapneumovirus, n = 10) during the first 2 years of observation, and therefore not included in the analysis. Viral densities, as estimated through cycle threshold values on nasopharyngeal swab polymerase chain reaction analyses, were not considered during the study. Higher pathogen densities may influence clinical outcomes.^20^ RISC scores could be derived for HIV-uninfected children only, as the parameter of clinical stage of HIV infection was not collected in our study. Children who were HIV-exposed uninfected were analyzed together with those who were HV-unexposed uninfected. This was due to not having exposure data available. Finally, although this was a similar finding in other studies, the small number of children hospitalized with RSV/SARS-CoV-2 coinfection limited the depth and degree to which the patients in this group could be analyzed.

## CONCLUSION

Coinfection with RSV/SARS-CoV-2 was an uncommon finding among children 0–59 months old hospitalized with severe LRTI in South Africa, which contrasts with the relative abundance of RSV-only and SARS-CoV-2–only LRTI. Children with RSV/SARS-CoV-2 coinfection had a higher prevalence of severe LRTI than those with RSV or SARS-CoV-2–only. Among HIV-uninfected children, RSV infection was independently associated with high RISC scores. These findings reinforce the urgent need for safe and effective SARS-CoV-2 and RSV vaccines, especially targeting children with comorbidities, and those living in LMIC, where the burden of disease is highest and access to medical resources are limited.
